# Transactions of Illinois State Medical Society

**Published:** 1876-01

**Authors:** 


Transactions of Illinois State Medical Society, 1875. Book pp. 288.

The reports on Insanity, Whooping Cough, Transfusion of Blood, Practical Medicine, Theory and Practice, President's Address, Hernia, Shortening in Fracture, Gynæcological Instruments, Obstetrics, Phthisis, Small Pox, all contain some points of interest.

